# μ-FTIR Reflectance Spectroscopy Coupled with Multivariate Analysis: A Rapid and Robust Method for Identifying the Extent of Photodegradation on Microplastics

**DOI:** 10.1021/acs.analchem.4c04281

**Published:** 2025-02-06

**Authors:** Eleonora Conterosito, Maddalena Roncoli, Chiara Ivaldi, Marysol Ferretti, Beatrice De Felice, Marco Parolini, Stefano Gazzotti, Marco Aldo Ortenzi, Valentina Gianotti

**Affiliations:** † Department of Sustainable Development and Ecological Transition, Università del Piemonte Orientale, Piazza Sant’Eusebio 5, Vercelli 13100, Italy; ‡ Department of Environmental Science and Policy, Università degli Studi di Milano, Via Celoria 2, Milano 20133, Italy; § LaMPo, Department of Chemistry, Università degli Studi di Milano, Via Festa del Perdono 7, Milano 20122, Italy

## Abstract

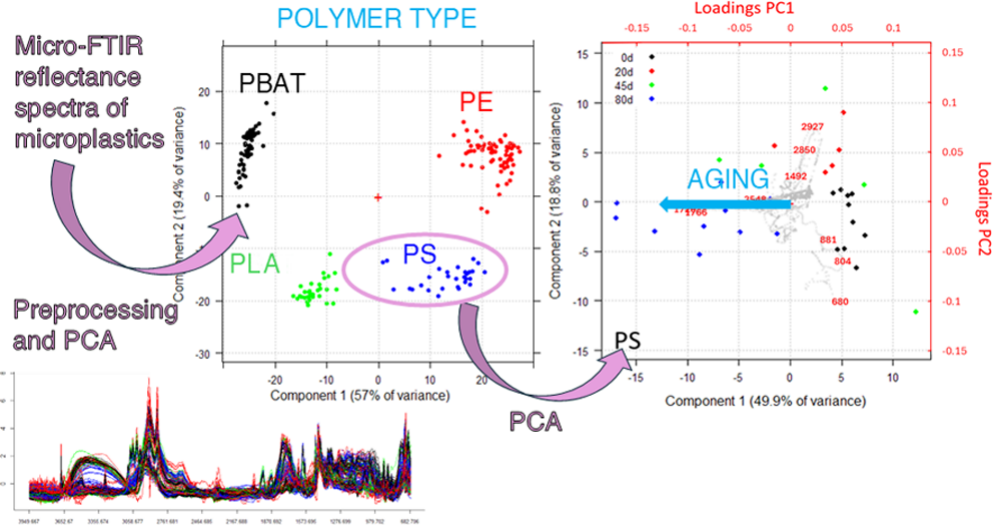

Understanding the origins of microplastics (MPs) and evaluating the consequences of plastic pollution require precise chemical information. Moreover, MPs undergo chemical changes due to photoaging, which are worth investigating since they can influence the effects of MPs on living beings and the environment. Micro-Fourier-transform infrared (μ-FTIR) spectroscopy is a key technique for screening MPs, combining optical imaging with chemical information from IR spectra. While reflectance μ-FTIR spectroscopy’s sensitivity to particle thickness and photodegradation complicates automated spectral matching, it can provide valuable information if coupled with multivariate analysis of the data. This study developed a robust method for identifying MPs, even when they are modified by photodegradation. Various acquisition methods (ATR-IR and μ-transflectance-IR), data pretreatments, and data set analysis procedures were examined, and critical aspects were addressed. The proposed method, using μ-TR-IR and principal component analysis (PCA), proved effective for classifying MPs and analyzing their degradation, offering increased sensitivity and a faster workflow compared with manual spectral comparison. μ-TR-IR showed earlier changes in relevant bands, indicating higher sensitivity to degradation than ATR-IR spectroscopy. Despite the notorious issue of spectral artifacts, our results suggest that valuable information can be collected without using sophisticated preprocessing techniques. On the contrary, the presence of the artifacts allows extracting some information on the particles’ thickness. Finally, PCA results were successfully validated for the polymer classification reliability by a test set and compared with the carboxyl index (CI) method to validate the ability to assess degradation. While CI is the most diffused parameter to assess polymer degradation, PCA, which considers the entire spectrum and does not rely on manual integration of single peaks, is inherently more robust than CI and can take into account multiple degradation mechanisms.

Microplastics (MPs) are defined as particles of various shapes and a wide granulometric distribution (from 1 μm to 1 mm) composed of different polymeric materials^1^ characterized by a variety of surface and bulk characteristics. In fact, they derive from complex generation mechanisms (physical, chemical, and biological) that result in widely different physicochemical properties that change with aging.^2,3^ Moreover, the small sizes facilitate their diffusion and transportation between air,^4^ water,^5,6^ soil,^7^ and biota, thereby promoting their ubiquity and interactions with other pollutants and microorganisms.^8,9^ Anyone studying the origin, fate, and impact on the ecosystem of MPs requires their precise and accurate identification and measurement. In this context, the most employed technique for the analysis of MPs is optical microscopy. This method is straightforward but highly time-consuming, strongly dependent on the analyst’s expertise, and does not provide chemical information about the material. For these reasons, nowadays microscopy is often coupled with spectroscopy. Both infrared (IR) and Raman spectroscopy are used since they are complementary, due to their different fundamental principles and the vibrational modes they detect.^10,11^ Raman spectroscopy can measure dark and thicker particles and offers spatial resolution down to 1 μm particle size, but this feature is seldom exploited due to the longer preparation procedure and measurement time.^12^ However, its use is often hindered by fluorescence from the sample or the surrounding material and is more affected by the substrate on which the particles are analyzed. In contrast, in IR spectroscopy, the main interference is caused by water.^11^ Among FTIR spectroscopy techniques, Attenuated Total Reflection (ATR) IR spectroscopy gained popularity thanks to the possibility of measuring solid samples, such as MPs, with minimal preparation and without limits to the sample thickness. ATR-IR spectroscopy is also available in a “micro-setup” where the ATR crystal is mounted on a micro tip that can be pressed on single particles on the sample holder to obtain a spectrum. Conversely, μ-FTIR spectroscopy in reflection/absorption reflection, commonly referred to as reflectance mode or transflectance (called μ-TR-IR from now on), offers the advantage of being a noncontact technique, avoiding the risk of crushing the MPs, especially the highly weathered ones, and allows faster measurements.^13,14^ μ-TR-IR in the medium infrared range uses a focalized IR beam with a resolution of a few tenths of μm, allowing the simultaneous optical and IR mapping of the sample.^13^ In contrast, μ-TR-IR is sensitive to the thickness and surface characteristics of the particles that add prominent features to the spectra. In fact, the IR radiation from the source is sometimes partially reflected by the sample surface, creating dispersion artifacts.^15^ The issue of spurious signals in the spectra of MPs recorded by μ-TR-IR has been already observed by Willans et al.^16^ and investigated by Romeo et al.^15^ and by Bassan et al.^17−19^ Preprocessing methods, such as extended multiplicative signal correction (EMSC) and mie extinction-EMSC, were developed to address the scattering and reflection issues, but their application on heterogeneous data sets comprising different polymers (such as real MPs data sets) is hindered by their need for a reference spectrum for the correction.^19^ The artifacts can interfere with the material identification by spectral matching with libraries, but on the other hand, they may contain information

Another important challenge for the spectroscopical study of natural MP samples, especially those found in water, is the issue of their separation from sediment and the removal of the organic layer, which often covers their surface, impacting the recorded spectrum. It would be better to eliminate these issues before spectrum recording. Separation from clay or sediment can be achieved through density separation,^20^ while various kinds of acidic, basic, or oxidant digestion have been investigated to remove the surface contaminants.^10,21^ Unfortunately, these chemical treatments could alter the MPs and their spectra; therefore, particular care has to be taken.

The degradation of MPs causes physical and chemical changes in the material and can have an impact on their biological effects and is therefore worth considering.^22^ The changes in the spectra can vary from very intense, representing a challenge for the spectral matching,^2^ to almost not detectable by the human eye. Therefore, the data analysis has to be aided by chemometric or machine learning methods.^23,24^ A study about the multivariate analysis on the photochemical aging of microplastic materials is reported by Zvekic et al.^25^ They used ATR-IR spectroscopy and melted the plastic pellets into flat disks (0.22–0.28 cm thick) to obtain better contact between the ATR crystal and the sample, thereby eliminating artifacts due to irregular surface features and avoiding distorting the pellets during the measurement. In a real-world sample, this approach is hindered by the chemical heterogeneity of microplastic particles, necessitating each particle to be measured separately. μ-ATR could be used, but it still needs contact and pressure between the instrument and particle, while μ-TR-IR in reflectance allows faster measurements without the need for pressing the particle and the risk of crushing it. Moreover, reflectance measurements provide greater penetration depth compared to ATR, where only the outer layer is measured.^26^ Carbonyl index (CI) calculation on FTIR spectra is currently one of the most used methods to assess the oxidation degree of polymers. Initially developed for polyolefins but later extended to be used on other polymers,^27,28^ it relies on the determination of the ratio between the area of the carbonyl peak and a reference band in the spectrum. This approach has the limitation of not being standardized^29,30^ and is particularly difficult to apply to polymers having carbonyls in their structure, producing mixed results.

The goal of the present work is the development of a comprehensive and robust method capable of identifying different MPs and elucidating the extent of their degradation. Spectra acquisition, data pretreatment, and data set analysis have been considered to achieve a method applicable to real unknown samples with the least possible number of steps and handling. For this purpose, a challenging set of samples was employed to test different acquisition and processing routes to study the performance of the method and the possible culprits. Four types of MPs, polyethylene (PE), polystyrene (PS), and the compostable polymers polylactic acid (PLA) and polybutylene adipate terephthalate (PBAT), were obtained from common objects to be representative of the materials that could be found in the environment. In particular, PBAT was obtained from common compostable bags used for shopping and disposal of wet organic waste. In Italy, where the law mandates that such bags be compostable, in compliance with EN 13432, the bags are made from a blend patented by Novamont, consisting of PBAT, thermoplastic starch (TPS), and other additives. The MPs were analyzed during their photoaging in a solar simulator. μ-TR-IR was chosen as the proposed technique, and ATR-IR spectroscopy was employed as a reference. Finally, the entire method was validated both for the identification capability and for the evaluation of photooxidation in comparison with the Carbonyl Index results.

## Materials and Methods

### Sample Preparation Methods

MPs samples were obtained by different cycles of freezing (in liquid nitrogen) and grinding in a PULVERISETTE 11 knife mill (Fritsch GmbH, Idar-Oberstein, Germany) from pieces of polymer cut from commercial products. PLA and PS were obtained from white single-use knives, so they likely contain TiO_2_ as a pigment, while PLA possibly also contains silicates as an additive. PE was obtained from a green chewing gum container, probably containing a green phthalocyanine dye. PBAT is Mater-Bi© by Novamont, a blend composed of PBAT, TPS, and additives. It was obtained from common compostable bags used for shopping and disposing of wet organic waste. The bag we used had a slight greenish color. The obtained material was then sifted to separate the fraction with size <1 mm for PBAT and PE, and with size <75 μm for all four materials. Accelerated photoaging of the MPs was obtained by UV irradiation treatment performed using a solar simulator apparatus (Solarbox, Co.Fo.Me.Gra, Milan, Italy) consisting of a Xe lamp chamber. Treatment conditions were chosen according to ASTM D3424. The Xe lamp was equipped with a filter simulating outdoor conditions, and the intensity was set to 500W/m^2^. The temperature was kept fixed at 50 °C through ventilation. The MPs were distributed across the bottom of a quartz bottle, limiting overlapping. Samples were analyzed after 20, 45, and 80 days of aging in the solar simulator, obtaining an accelerated aging factor 24 times (80 days correspond to about 5 years)

### Spectroscopy and Microscopy Techniques

μ-TR-IR measurements were performed on MPs deposited on silver metal membrane filters (Sterlitech; pore size 0.8 μm, Ø = 13 mm) using a Nicolet iN10 MX Infrared Imaging Microscope (Thermo Scientific, Waltham, MA, USA) controlled by OMNIC Picta software (Thermo Scientific, Waltham, MA, USA) in reflectance mode. Focusing was performed in spectral preview mode, 256 scans (range 4000–675 cm^–1^) were acquired for each spectrum with 4 cm^–1^ resolution, and Beer-Norton apodization applied.

ATR-IR analyses were carried out by using a Nicolet iS50 FTIR (Thermo Fisher Scientific, Waltham, MA, USA) spectrometer. A total of 64 scans were acquired for each spectrum, in the range 4000–400 cm^–1^ with 4 cm^–1^ resolution. Scanning electron microscopy (SEM) images were recorded on a Phenom Pro Desktop (Thermo Fisher Scientific, Waltham, MA, USA) microscope. The samples were coated with a thin gold layer to ensure surface conductivity.

### Data Processing and Statistical Analysis

Prior to multivariate analysis by Principal Component Analysis (PCA), all spectra were preprocessed using Spectragryph.^31^ The spectra, expressed in Log(1/R) to facilitate the interpretation of PCA results,^32^ were cut in the region 3949–675 cm^–1^. The preprocessing steps described hereafter were applied in different combinations, following a systematic approach, as described in the results and discussion section. Baseline correction was performed using the adaptive algorithm on the individual spectra with the coarseness set to 35 and zero offset. This coarseness value was chosen based on a visual assessment of the spectra before and after subtraction. Scattering correction was performed by using the standard normal variate (SNV) algorithm. Smoothing was performed using the Saviztky–Golay algorithm with a window size of 9 points and a third order polynomial. The preprocessed spectra were then imported in CAT (Chemometric Agile Tool)^33^ to perform PCA.

A test set made up of 11 spectra of LDPE particles, 10 spectra of PS particles (from objects different from those in the training data set), and 6 spectra each for PP and PET particles was used to validate the PCA results.

The carbonyl index (CI) was calculated on all μ-TR-IR spectra according to the SAUB method.^30^ The areas underlying the peaks were integrated using the zero baseline option in Spectragryph. The CI was then calculated on each spectrum of the complete data set as the ratio between the area of the carbonyl peak between 1850 and 1650 cm^–1^ and the reference peak (methylene CH_2_ scissoring) between 1500 and 1420 cm^–1^. For each single polymer type, CI values were calculated as follows:

CI_PBAT_ = A_1850–1650_ /A_1492–1430_^27^

CI_PE_ = A_1808–1660_/A_1500–1450_^30^

CI_PLA_ = A_1760–1851_/A_1422–1485_^27^

CI_PS_ = A_1850–1650_/A_885–850_^34^

## Results and Discussion

With the aim of developing a complete method that includes the least number of steps and handling, a set of MPs encompassing the difficulties of real analysis was chosen. Consequently, MP samples derived from four types of polymers (PBAT, PE, PLA, PS) underwent accelerated photoaging in a solar simulator for different lengths of time. All materials were sourced from common commercial products to better simulate plastics found in the environment. Particle sizes for PLA and PS were <75 μm and were easily obtained through milling. Milling PBAT and PE was more challenging. Therefore, for PBAT and PE, two fractions with different sizes were separated and examined (<75 μm, <1 mm), as it is reasonable to assume that natural MPs made of these materials could also be of larger sizes.

Approximately 10 particles were analyzed for each sample, resulting in a data set composed of 192 μ-TR-IR spectra. When measuring the spectra, particular care was given to the focusing since it is necessary for improving the spectrum quality. In detail, the focusing was performed by visualizing the spectrum in real time (in preview mode). This is essential to avoid saturation and ensure that the IR beam is focused. On the obtained data, multivariate pattern recognition analysis of Principal Component Analysis (PCA) coupled with an optimal and robust data preprocessing method was applied.

### Preprocessing Optimization

The aim of preprocessing optimization was to identify a procedure that minimally alters the spectra, allows optimal extraction of relevant information through PCA, and can be automatically applied to the entire data set. Figure S1 reports some examples of the data set spectra before any correction, showing that they have very different baselines and different global intensities. The whole data set was used without manually rejecting any spectrum, even if affected by evident artifacts. To investigate the effect of different preprocessing procedures, baseline correction, scattering correction, and smoothing were evaluated. The algorithm adopted for each procedure was optimized in a preliminary phase. The optimal conditions are detailed in the Materials and Methods section. For scattering correction, SNV normalization was chosen over EMSC since it can be applied to the whole data set without the need for a reference spectrum. Since the preprocessing methods can be applied alone or associated, six combinations were applied to the whole data set and explored systematically (Table 1).

**Table 1:** Systematic Evaluation of the Preprocessing

Sequence	Baseline	Scattering	Smoothing
1	no	yes	no
2	yes	no	no
**3**	**yes**	**yes**	**no**
4	yes	yes	yes
5	no	yes	yes
6	yes	no	yes

The preprocessing step order was selected based on experience (baseline first, then scattering correction, and smoothing last) and kept fixed. PCA was then applied to each data set to evaluate the impact of preprocessing on information extraction.

PCA is a multivariate analysis technique used to reduce the dimensionality of a data set. In the present case, the data set is composed of IR spectra, and each spectrum represents a sample, while each wavenumber represents a variable. By examining the score plots of the PCA of the six data sets (Table S3) it appears that baseline subtraction or scattering correction alone does not provide a good separation of the samples (#1 and #2), even with smoothing (#5 and #6). Preprocessing sequences #3 and #4 allow obtaining the best separation of the samples according to the polymer type, demonstrating that baseline and scattering correction are both necessary. Preprocessing sequence (no. 3) was chosen as optimal since the addition of the smoothing step (no. 4) does not improve the separation of the samples.

### PCA Analysis on the Complete Data Set

**Figure 1:**
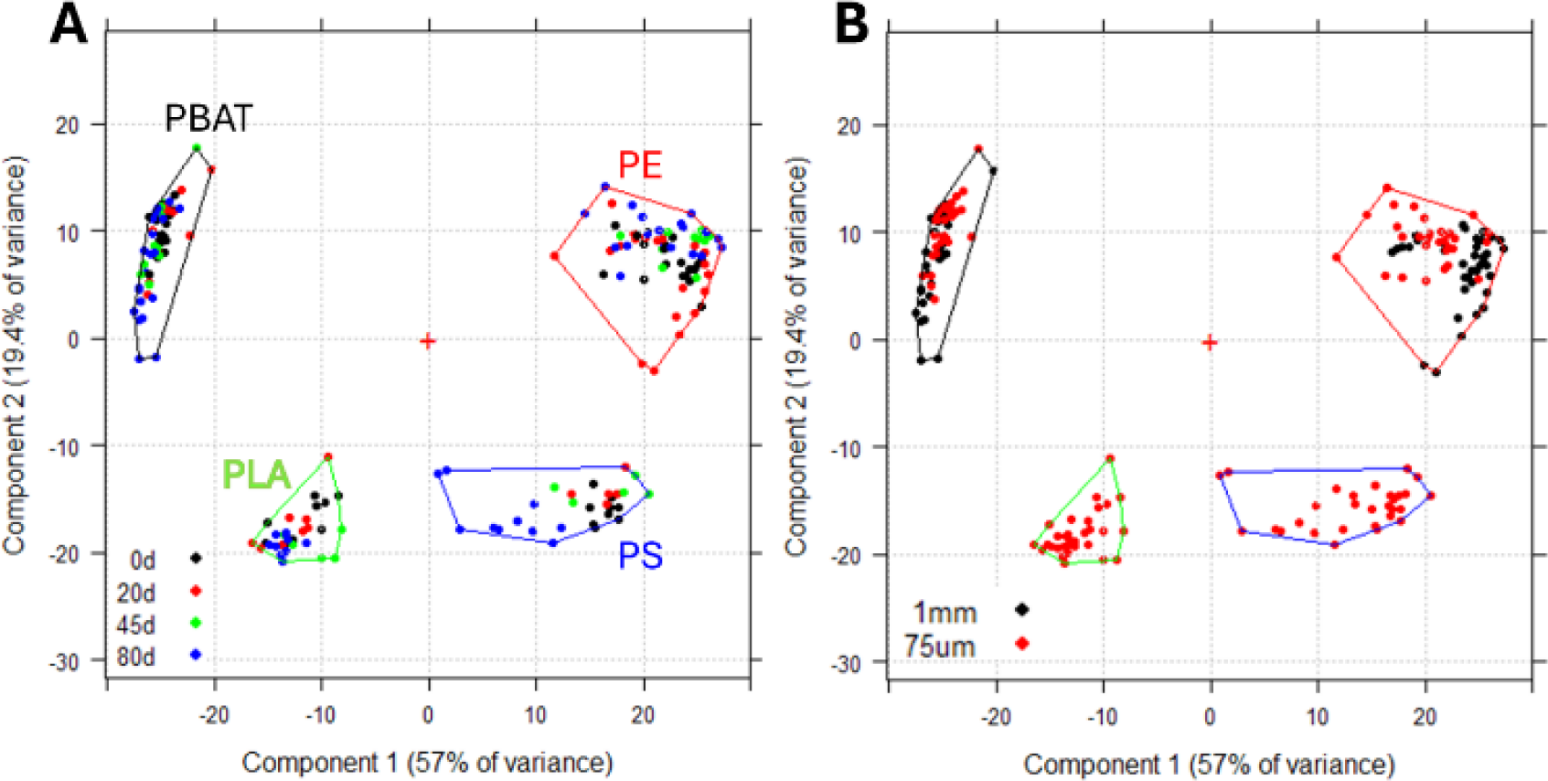
PCA scores plot for the complete data set. Convex hull polygons were plotted to include all samples of the same polymer. (A) The points, representing the samples, are color coded according to the aging time in days. (B) Samples are brushed according to size.

PCA performed on the complete data set with the optimal preprocessing sequence (#3) provides the scores plot of Figure 1A in which PC1 and PC2 clearly separate the samples into four groups corresponding to the four polymers under study.

PBAT samples fall closely together, while PE samples are the most dispersed ones. PLA samples demonstrate a clear separation based on aging time, whereas PS samples aged 80 days are separated from the others. PBAT data points fall closely together and do not show any clear trend attributable to aging, suggesting that PBAT MPs are quite stable to photo-oxidation, as already observed in the literature when MPs were irradiated in dry and sterilized conditions, such as in a solar simulator.^35^

Comparing the score plot with aging color code (Figure 1A) with the one brushed according to the size of the samples (Figure 1B), it is possible to see a partial separation according to particle size in the case of PE, while no such separation is observed in PBAT.

SEM images (Table S2) confirm that PBAT and PE particles are characterized by different morphologies. PBAT particles have one dimension significantly shorter than the other two, so they lie flat on the filter and present a similar thickness in the direction of the IR beam. In contrast, PE particles exhibit more irregular morphologies and lie randomly on the filter, resulting in varying thicknesses correlating directly with the size. If the thickness of the particle exceeds the IR beam penetration, artifacts are formed because the reflection from the surface of the particle is the prevalent signal.^15,17^ The spectra of thicker PE particles are, in fact, affected by total absorption/reflection artifacts in the CH stretching region (2600–3000 cm^–1^).^16^ In the data set, some spectra measured on thicker particles (mainly aged 20 days) exhibiting these spectral features were included on purpose (an example of one of the worst cases is reported in Table S1). These spectra were misclassified or classified as PE with a match below 40% by spectral matching, while PCA correctly classifies them as PE.

**Figure 2:**
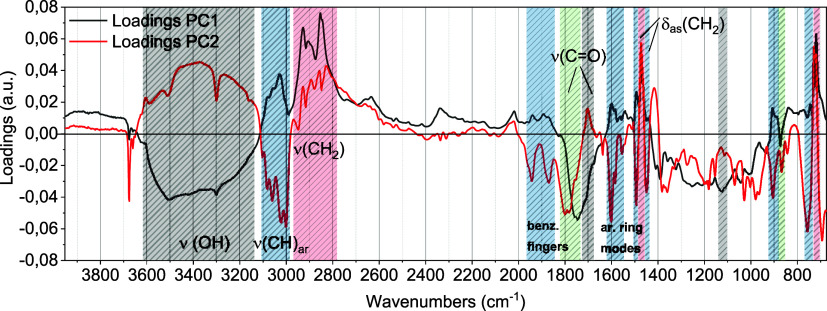
Loadings plot of the PCA on the complete data set. The most relevant wavenumbers determining the position of samples are highlighted: red = PE; blue = PS; black = PBAT; green = PLA.

The loadings plot (Figure 2) allows for associating the samples’ positions with the variables that characterize them, i.e., to identify the most relevant spectral bands for the description and grouping of the samples (Figure 1). PE samples are positioned in the top right quadrant in Figure 1 and are therefore described by the regions with positive loadings of PC1 and PC2 (highlighted in red) in Figure 2.

Similarly, PS is characterized by the bands highlighted in blue (with positive PC1 loadings and negative PC2); PBAT is characterized by the gray bands (negative PC1 loadings and positive PC2); while PLA is characterized by the green bands (negative loadings of both PC1 and PC2). These bands (Figure 2) are consistent with the attributions made on ATR-IR spectra (Figure S2), assigning them to the relevant functional groups of each polymer. Notably, PS is placed at positive PC1 and negative PC2 values by the bands attributed to the aromatic (ar) groups (ar. CH stretching in the 3000–3100 cm^–1^ region, benzene “fingers” in the 1800–2000 cm^–1^ region, and ar. ring modes at 1600 cm^–1^). PBAT and PLA are both found at negative PC1 values since they contain carbonyl groups in their structure and therefore have a C=O stretching band centered at 1740 cm^–1^.

### Method Application on the Test Data Set

**Figure 3:**
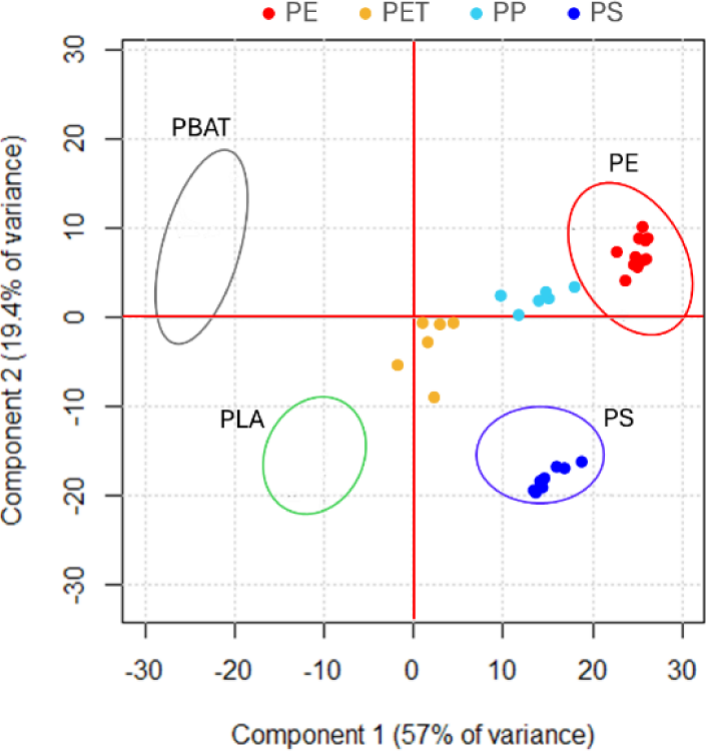
Scores plot showing the position of the test set sample (circles) on the train set PCA (ellipses).

At last, the entire optimized method was applied to a spectral data set collected on different MPs to test its applicability and robustness. A test set, composed of PS, PE, PP, and PET, was preprocessed according to the optimized sequence (no. 3) and then projected onto the PCA performed on the training set. In Figure 3, the points indicate the position of the test set samples, while the ellipses indicate the position of the training set samples.

The positions of the test set samples show that PE and PS are placed in the correct position as they are superimposed on the same polymer from the training set.

The PP samples are located close to the PE samples from the training set, which is reasonable given the chemical similarity and shared spectral features of these two polymers; however, they remain distinct from PE.

Despite PET posing the challenge of featuring a terephthalate group like PBAT, it is well separated and placed near zero at slightly negative values of PC2. This is probably because our PBAT is a thermoplastic starch/PBAT blend and shows a large OH band in the region between 3000 and 3600 cm^–1^ due to the presence of starch, which makes it very different from PET. Moreover, considering the typical spectral features of pure PBAT and PET, we can see that PBAT shows bands at 1123 and 937 cm^–1^, evidenced by negative loadings of PC1, that are not present in the PET spectra and contribute to their separation.^36^

### Degradation Evaluation

As seen in Figure 1, PCA performed on the entire data set shows trends related to the photoaging time, demonstrating its capability to discriminate samples with different aging. Here, PCA is replicated on each polymer separately to see in more detail the separation of the samples according to aging time, correlating it to the changes in the spectra.

The expected variation due to photodegradation in the IR spectra of polymers that underwent photooxidation^37^ typically occurs in the carbonyl stretching mode region (1800–1700 cm^–1^).^38,39^ Specifically, for polymers containing carbonyl groups prior to the degradation, a broadening in the carbonyl band should be detected due to the formation of different carbonyl-containing species (aldehydes, ketones, lactones, carboxylic acids, etc.) or an increase in disorder due to the formation of amorphous regions.^40^ In contrast, polymers devoid of such functional groups exhibit an increase in this signal due to the formation of carbonyl species following Norrish-type degradation processes.^41^ Other significant changes in the IR spectra upon degradation are typically observed in the OH stretching region (3600–3200 cm^–1^), with an increase in the intensity of this broad signal due to the formation of hydroperoxides from the reaction with oxygen and their subsequent photolysis, which generates hydroxyl groups.^25^

**Figure 4:**
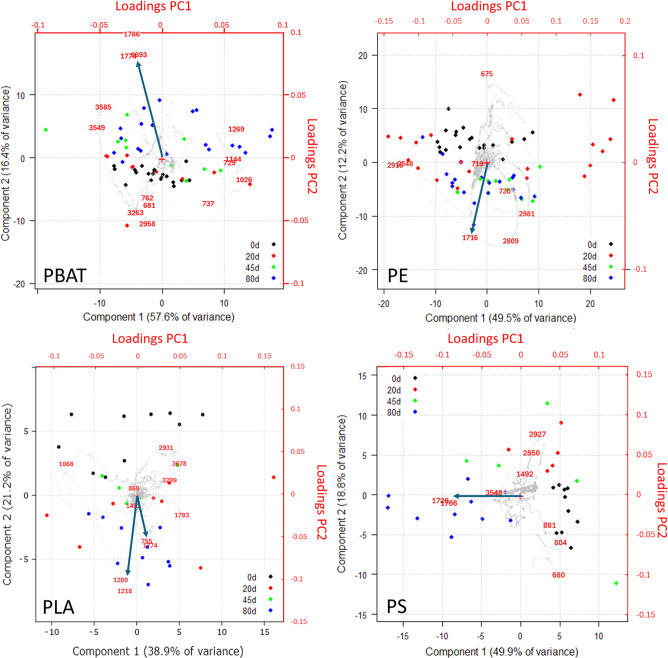
Biplot of PCA performed on each polymer separately. The colored dots represent the scores of samples aged 0 days (black), 20 days (red), 45 days (green), and 80 days (blue). The gray dots represent the variables’ (wavenumbers) loadings. The red labels highlight the most significative wavenumbers, and the arrows show the direction pointing the variables explaining aging.

The biplots of Figure 4 (scores and loadings plot grouped on the same graph) allow us to determine, for each polymer separately, if a trend according to aging time is visible and, at the same time, which wavelengths are responsible for the separation of the samples according to their aging time (i.e., the bands changing intensity due to photooxidation).

**Figure 5:**
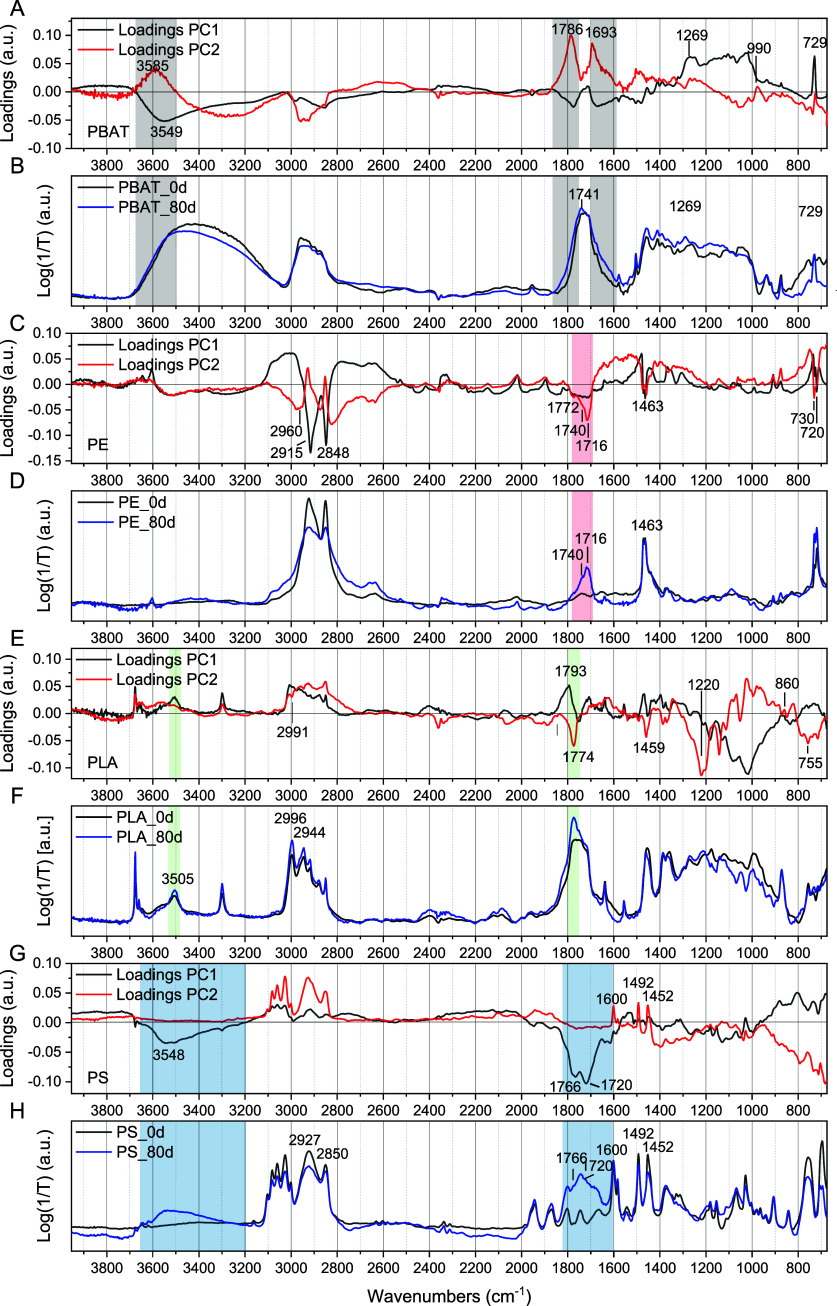
Plot of the PCA loadings of PC1 (black) and PC2 (red) of PBAT (A), PE (C), PLA(E), and PS (G) samples, respectively, and representative μ-TR-IR spectra of PBAT (B), PE (D), PLA (F), and PS (H) collected after 0 (black) and 80 days (blue) of aging.

In each biplot, an arrow points in the direction of the variables explaining most of the aging process, and the wavelengths with high loadings are highlighted in red. As an example, by observing the PC1 versus PC2 scores of the PBAT samples, it is evident that the aging is explained by PC2. In fact, most samples with a long aging time fall at positive values of PC2, meaning that the wavelengths with high loadings on PC2 increase their intensity with aging. The opposite occurs for the wavelengths placed at negative values on PC2, and they decrease their intensities upon aging. It is also worth noting that the trend of PBAT samples due to aging was not visible in the PCA of the full data set, but now, analyzing it separately, it has become evident. Figure 5, showing the loading plot alongside the real μ-TR-IR spectrum for each polymer, highlights the positions of the relevant wavenumbers extracted from the biplot.

Starting from PBAT, the informative wavelengths are placed at a positive value of PC2 and are evidenced in gray on the loadings plot (Figure 5A) and μ-TR-IR spectrum (Figure 5B). Most notably, two intense features, at 1693 and 1786 cm^–1^, that correspond to the shoulders of the carbonyl band, centered at 1720 cm^–1^ in the PBAT spectrum (Figure 5B) indicate that the carbonyl band becomes broader with photoaging since the intensity increases at the sides of the original band. This is consistent with the formation of different carbonyl species from the chain scission of the ester group through Norrish I type reactions.^42,43^ Namely, the band at 1693 cm^–1^is attributed to low molecular weight esters and the band at 1786 cm^–1^ to the free C=O group.^44^

Regarding PE, the aging trend is explained mostly by PC2, and the involved variables (Figure 4) correspond to a composite and intense negative band in the 1772–1716 cm^–1^ region in the loadings plot (Figure 5C). According to the literature, this IR band increases with aging, and it is attributed to the stretching of C=O groups in carboxylic acids (1716 cm^–1^), esters (1740 cm^–1^) and lactones (1772 cm^–1^). These changes are consistent with a Norrish-type degradation mechanism.^38,45^ A group of samples is isolated by PCA at the right of the biplot (Figure 4) due to the low intensity of the bands having high negative loadings of PC1 (at 2915 and 2848 cm^–1^) visible in Figure 5C, black curve. These are the already mentioned spectra affected by artifacts due to their thickness. It is important to point out that PCA correctly placed these samples in terms of aging, since they are characterized by positive values on PC2.

In the biplot of PLA, the variables correlating with aging have negative loadings on PC2. Specifically, the signal at 1774 cm^–1^ can be attributed to the C=O stretching of organic peroxide groups that are formed during the aging process.

In fact, these two bands appear as anticorrelated variables in the biplot of PLA (Figure 4). The band associated with crystallinity (755 cm^–1^) is instead correlated to the 1774 cm^–1^ band, associated with the formation of peroxide groups, indicating that the photooxidation of PLA results in both the formation of peroxide groups and an increase in crystallinity. The sharp band at 3676 cm^–1^ is attributed to the stretching of non-H-bonded −OH groups, while the broad band between 3500 and 3600 cm^–1^ is assigned to the OH stretching of water adsorbed by the polymer. This band is lower in the spectrum of samples aged 80 days due to water evaporation during the treatment inside the solar simulator. The bands at 1638 and 1555 cm^–1^ can be attributed to additives in the PLA.^46^

In the biplot of PS (Figure 4), aged samples are placed at high negative values of PC1 by the variables 1720 and 1766 cm^–1^. In the loadings plot of PS (Figure 5G), these bands have high negative loadings and are therefore responsible for the separation of samples with high aging. By comparing the spectra of samples aged 0 and 80 days, it can be seen that the latter have a strong signal in this region, which is attributed to the C=O stretching of carboxylic acids (1720 cm^–1^) and lactones (1766 cm^–1^). Moreover, the OH stretching band, centered at 3548 cm^–1^, appears in the aged samples. These changes are consistent with the photooxidation mechanisms of PS by Norrish-type reactions and hydroperoxide photolysis.^41^

**Figure 6:**
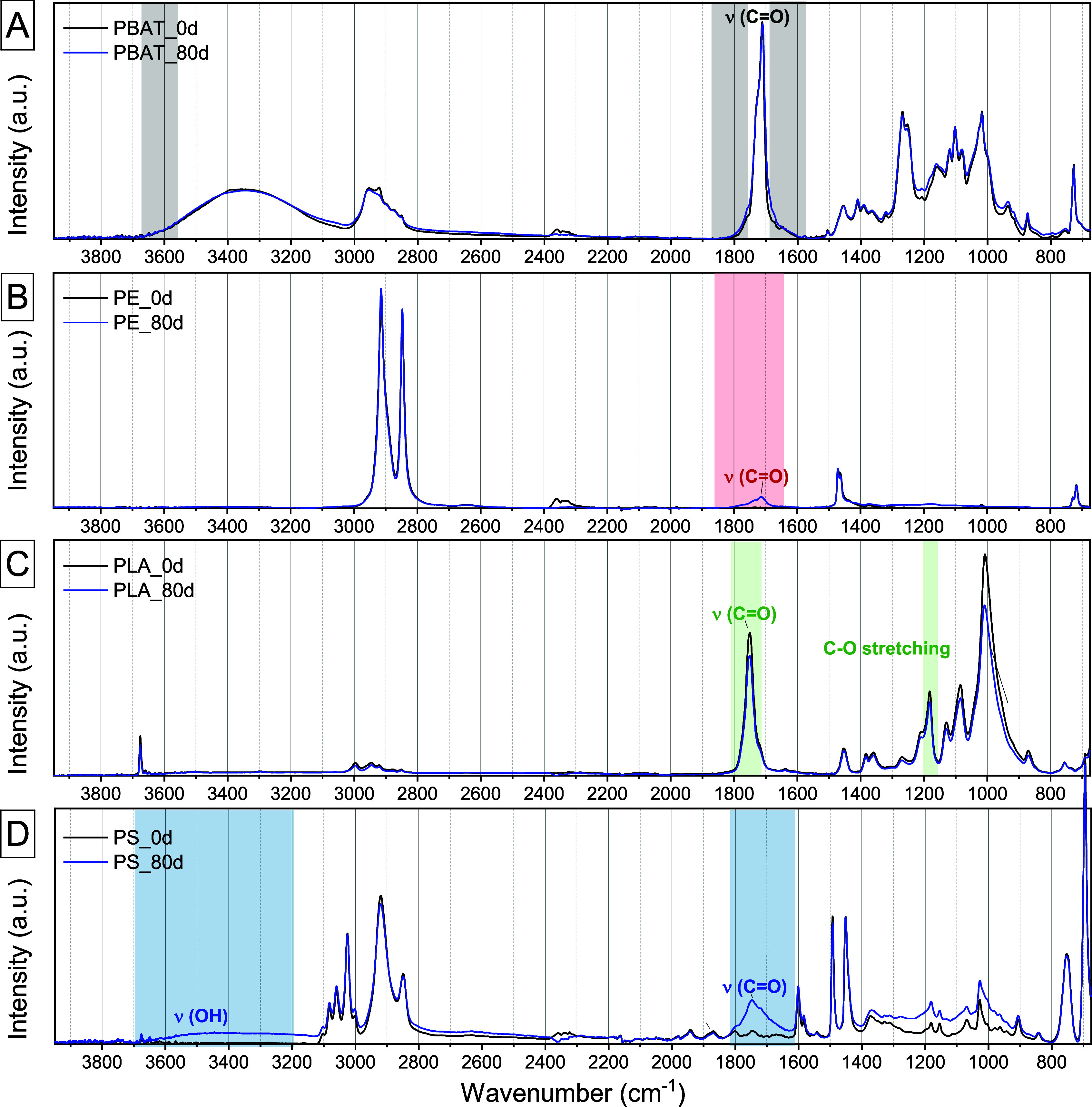
ATR spectra of (A) PBAT, (B) PE, (C) PLA, and (D) PBAT. Characteristic bands have been assigned for each spectrum. The same color codes as those in the previous figures are employed (black = PBAT; red = PE; green = PLA; blue = PS).

To validate the obtained data with the most employed technique, ATR spectra of the polymers before and after 80 days of photoaging treatment were collected (Figure 6). Appreciable changes in the carbonyl region can be detected only in the PE and PS ATR spectra (Figure 6B,D), while in the spectra of PBAT and PLA (Figure 6A,C), the small band broadenings promptly recognized by the PCA are hardly even visible. Similarly, variations in the OH stretching region are appreciated only in the PS spectrum, while they are totally absent in that of PBAT. These data highlight that PCA applied to μ-TR-IR data allows one to correctly assign samples based on their chemical composition, improving sensitivity and feasibility on real MPs with respect to ATR-IR.

### Degradation Extent Evaluation

The reference method to study the amount of degradation of polyolefins (also extended to other kinds of polymers) is the carbonyl index (CI) calculation. It relies on the calculation of the ratio between the IR band of carbonyl stretching (νC=O) and the reference band (usually CH_2_ stretching).

Despite the limitations of this index, also due to a non-standardized method, it has been widely adopted as a practical way to express the amount of degradation of polymers in a relative and semiquantitative way.^22,29,30^

**Figure 7:**
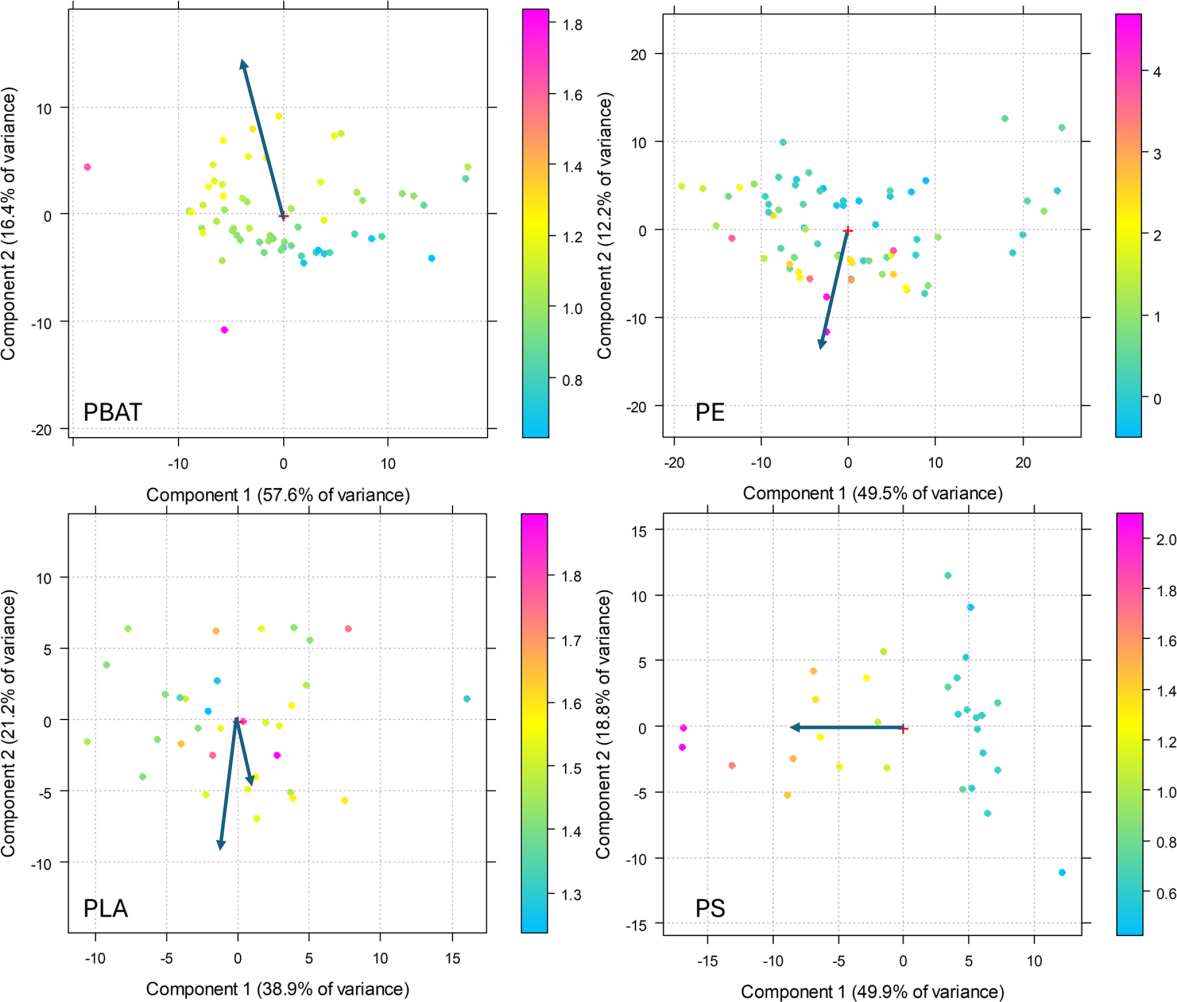
PC1 vs PC2 score plot of the PCA performed on each polymer, brushed according to the carbonyl index values.

By comparing the PCA results with the calculated CI, it is possible to perform validation of the proposed method. The CI values were calculated on each spectrum according to the SAUB method^30^ and employed to brush the PCA scores to evidence if they are in accordance with each other. In Figure S8, it is possible to appreciate the trends of CI, which are in good agreement with the trends shown in Figure 1. It is also evident that, as expected, the carbonyl indices have different ranges for each polymer since each polymer is characterized, before aging, by a characteristic ratio between the carboxyl and reference CH_2_ bending peak. Figure 7 shows the scores plot of the PCAs performed on MPs of each polymer separately, brushed according to the CI calculated from specific bands for each polymer, as described in the experimental section. The arrows point in the direction of the loadings highlighted in Figure 4, demonstrating that the CI aligns with the aging trend, as evidenced by the PCA.

In the case of PLA, the calculation of CI is complicated by the fact that the C=O stretching band is already present in the starting material, and the C=O groups of peroxides formed due to photooxidation appear as a shoulder of this strong band, making the calculation unreliable, even if the spectra clearly show changes attributable to degradation. In fact, in the literature, the CI of PLA is reported to decrease with aging,^28,47,48^ sometimes to increase,^27^ or to slightly decrease before slightly increasing.^3^

As mentioned, in the case of PLA, the PCA also highlights the band at 755 cm^–1^ which is related to the crystallinity of PLA. By calculating the ratio between the area of the band at 755 cm^–1^ and the band at 860 cm^–1^, form a “crystallinity index” can be obtained. This index is highlighted in the scores plot in Figure S9. The case of PLA clearly evidences one important advantage of PCA over the calculation of CI: PCA considers the entire spectrum rather than just one band, making the technique more sensitive and capable of considering different mechanisms simultaneously.

## Conclusions

A comprehensive and robust method capable of identifying different MPs and elucidating the extent of their degradation was developed. The proposed method is based on μ-TR-IR since it offers some advantages over μ-ATR-FTIR, such as higher sensitivity, the possibility to record simultaneously the optical image and spectrum, and the absence of contact between the instrument and the particle. Coupled with principal component analysis, μ-ATR-FTIR proved to be an effective method for the classification of MPs and subsequent analysis of their degradation, providing increased sensitivity with respect to the manual comparison of spectra, database spectral matching, and a drastically faster workflow.

Despite the notorious issue of spectral artifacts, which induce wrong classification or low figures of merit in spectral matching, it was shown that it is not always necessary to correct these artifacts by using sophisticated preprocessing techniques prior to multivariate analysis or to exclude the affected spectra to obtain a correct classification. Instead, the presence of the artifacts allowed for extracting information about the particles’ thickness. Finally, PCA results were successfully validated for the polymer classification reliability by a test set of MPs. After classification, the whole spectrum can be further used to obtain information about the extent of degradation through PCA, instead of relying on the comparison of specific spectral bands, such as for the CI calculation. PCA has the advantage of considering different simultaneous changes, and the bands affected by degradation-related changes do not have to be chosen a priori but are highlighted by the PCA itself.

Upon investigation of the optimal pretreatments for natural samples and their effect on MPs spectra, which will be addressed in a further study, the proposed method can be applied to samples of microplastics obtained from the environment since the proposed automation of the collection of the spectra opens the possibility of an almost completely automatic analysis of high numbers of spectra. In fact, the preprocessing can be applied in batch to all of the collected spectra and does not require preliminary information on the type of MP investigated. These aspects are necessary when dealing with natural microplastics whose composition and residence time in the environment are unknown.

## Supporting Information Available

SEM analysis details and images, additional PCA analyses, and FTIR spectra (PDF)
