# Supplementary material for: μ-FTIR Reflectance Spectroscopy Coupled with Multivariate Analysis: A Rapid and Robust Method for Identifying the Extent of Photodegradation on Microplastics
Source: Anal Chem. 2025 Feb 6;97(6):3263–73. doi: 10.1021/acs.analchem.4c04281 (PMC11840800; doi:10.1021/acs.analchem.4c04281)
Supplement: Supplementary file 1 — ac4c04281_si_001.pdf [file ac4c04281_si_001.pdf]

μ-FTIR reflectance spectroscopy coupled with multivariate analysis: rapid and robust method for identifying the extent of photodegradation on microplastics

Eleonora Conterosito[1], Maddalena Roncoli[1], Chiara Ivaldi[1], Marysol Ferretti[1], Beatrice De Felice[2], Marco Parolini[2], Stefano Gazzotti[3], Marco Aldo Ortenzi[3] and Valentina Gianotti[1]*

[1]Department of Sustainable Development and Ecological Transition, Università del Piemonte Orientale, Piazza Sant'Eusebio 5, 13100 Vercelli, Italy

[2]Department of Environmental Science and Policy, Università degli Studi di Milano, Via Celoria 2, 20133 Milano, Italy

[3]LaMPo, Department of Chemistry, Università degli Studi di Milano, Via Festa del Perdono 7, 20122 Milano, Italy

This file includes:

Supplementary IR spectra (Figure S1- S7)

Examples of raw spectra and corresponding particle image (Table S1)

Scanning electron microscopy details and images (Table S2)

Supplementary details and graphs of principal component analyses (Table S3, Figures S8, S9)

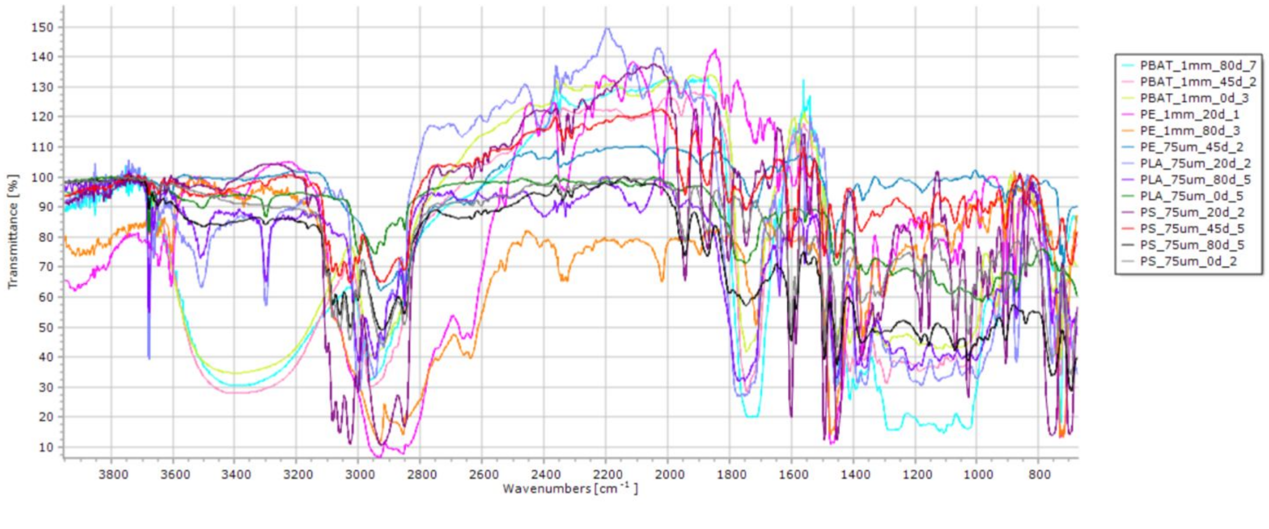

Figure S1: Some examples of the μ-TR-FTIR spectra before preprocessing.

Table S1 : Some examples of microscope images and corresponding μ-TR-FTIR spectrum

PS <75 t0	
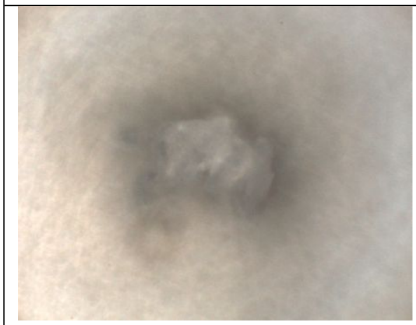	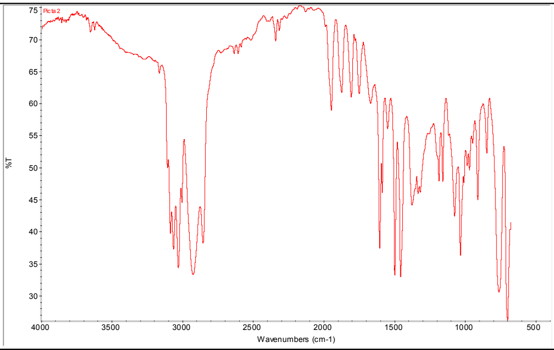
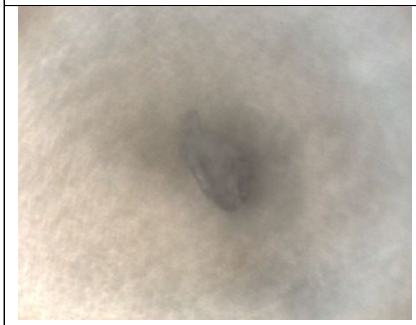	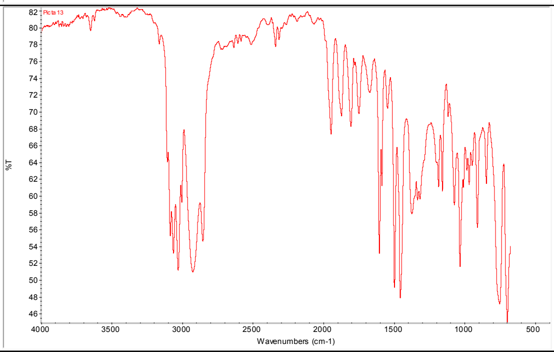
PE < 1mm t0	
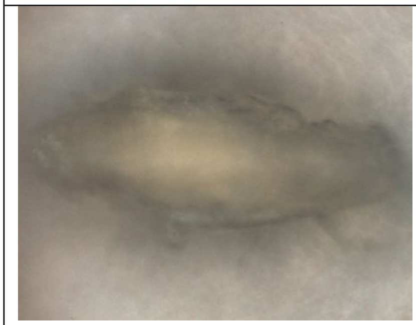	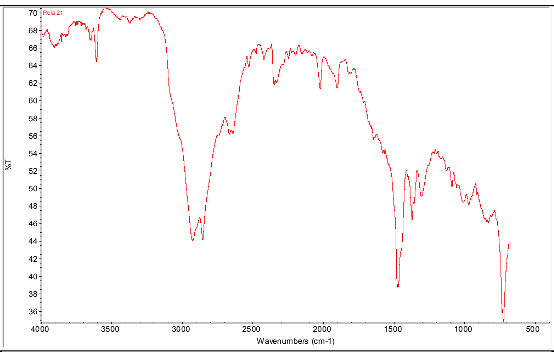
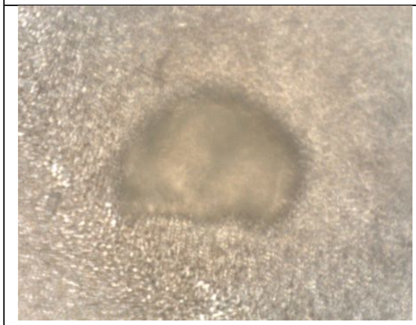	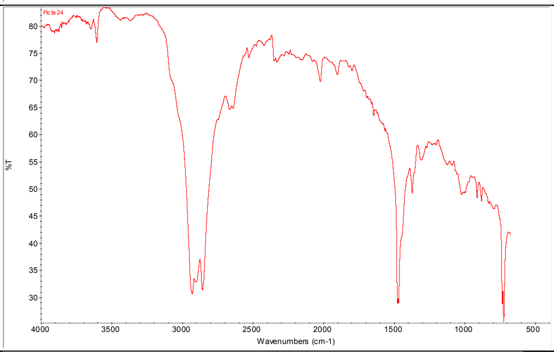
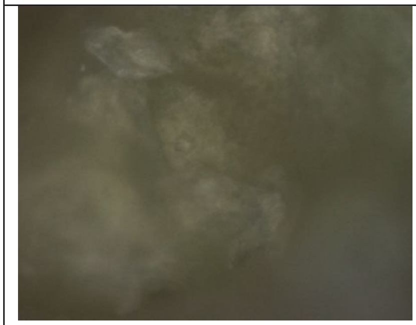 t = 20d	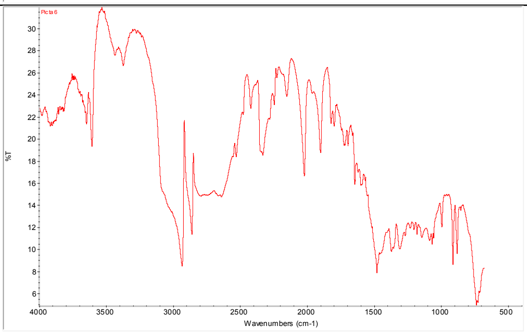 t = 20 d

PE <75um t0

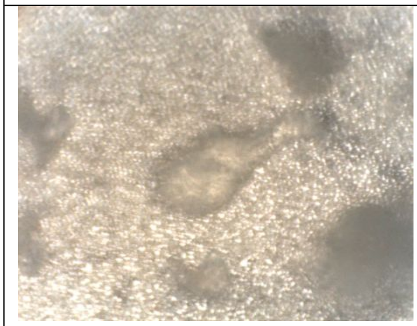

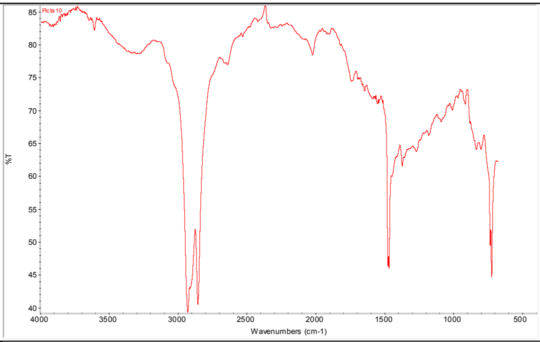

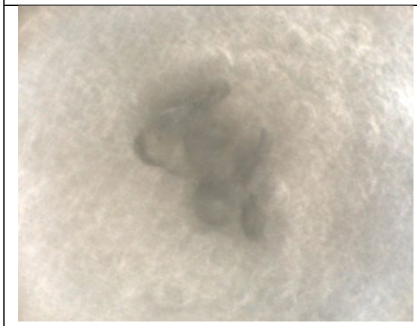

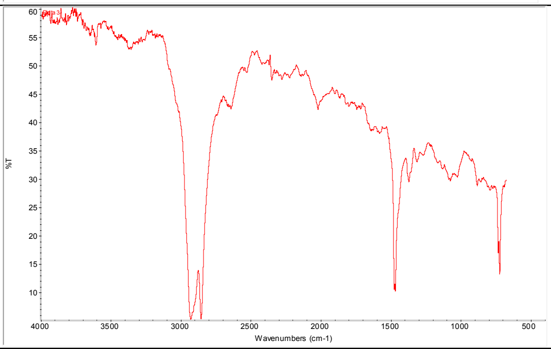

PLA <75um

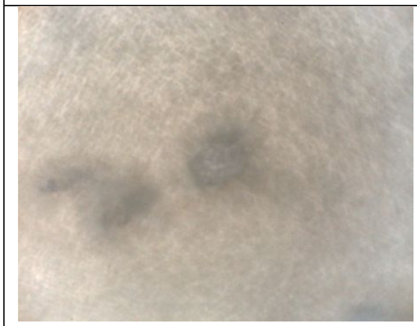

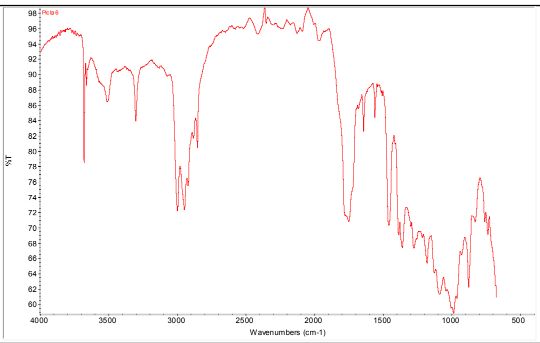

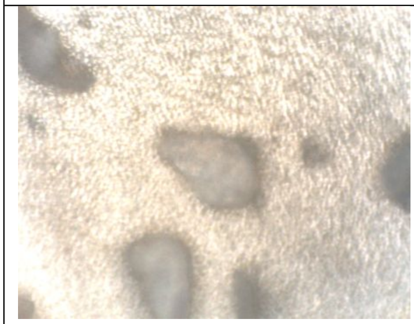

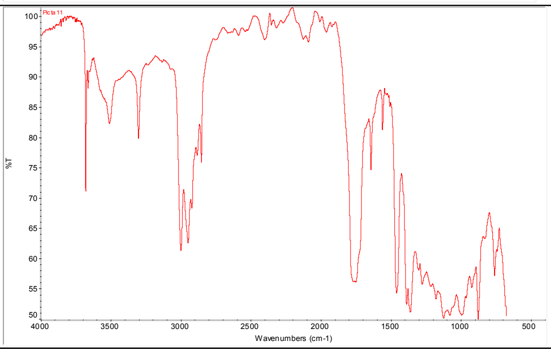

PBAT <75 um

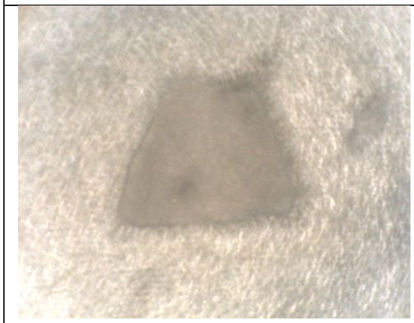

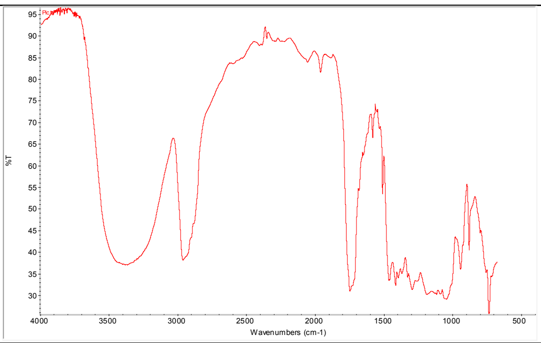

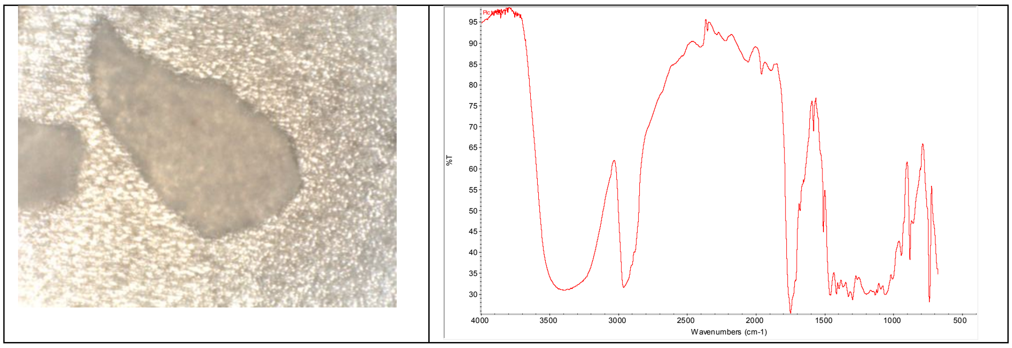

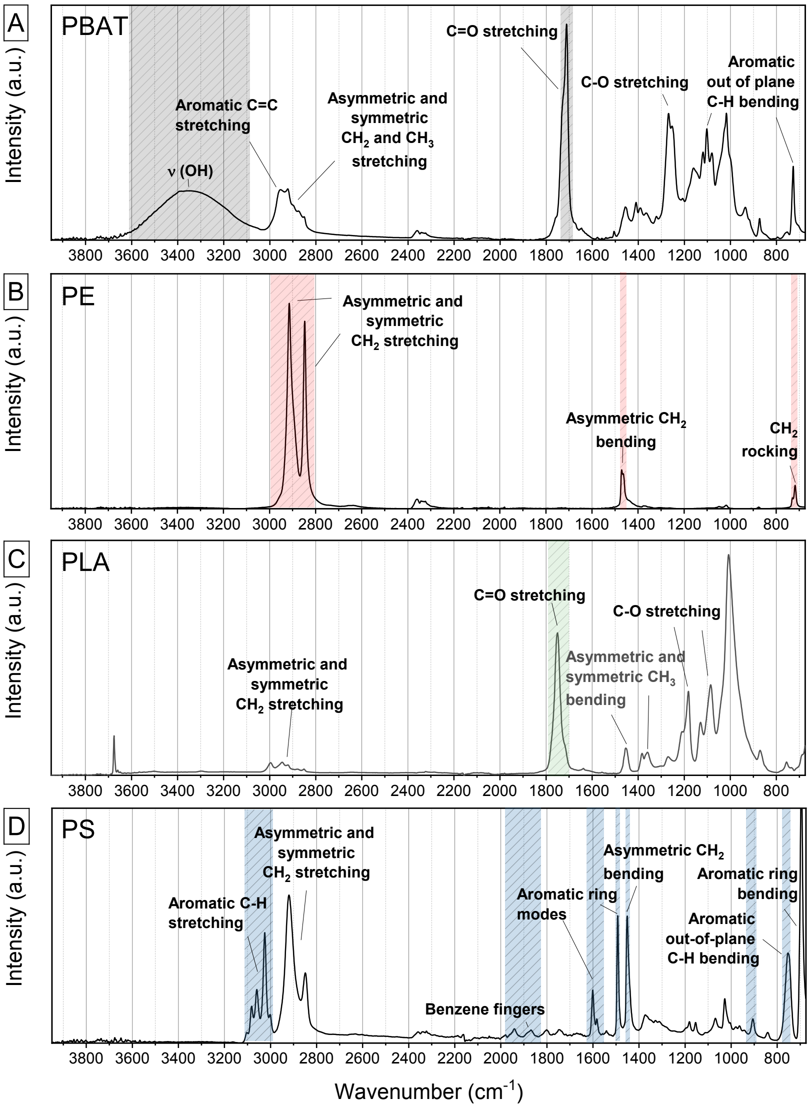

Figure S2: ATR-FTIR spectra with the characteristic bands of the polymers assigned.

SEM analysis

SEM images were collected on the microplastics before the aging and are shown in Table S1.

PBAT particles, obtained by milling a shopping bag, have a sheet like morphology and approximately all the same thickness (~10 μm). There is greater variability in the other two dimensions which are generally between 200 and 600 μm in the sample classified <1 mm and between 50 and 200 μm in the sample classified >75 μm.

PE particles exhibit heterogeneous and irregular morphology, comprising both rounded particles and frayed thinner pieces. The thickness of the frayed pieces is estimated to be around ~10 µm. In samples classified as <1 mm, the longer sides of the particles measure approximately 200-400 µm, while in samples classified as <75 µm they are about 100 µm with the exception of some very elongated particles that have one side above 700 µm.

PLA particles are quite uniform in size, with an average side length of 100 µm, and appear as rounded or flattened chunks of heterogeneous composition. The flat particles can be as thin as ~10 µm.

PS particles have a morphology and size distribution similar to PLA but they appear of uniform composition.

Table S2: Scanning Electron Micrography pictures of the samples.

PBAT<br>1mm	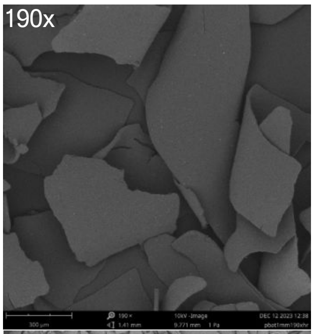	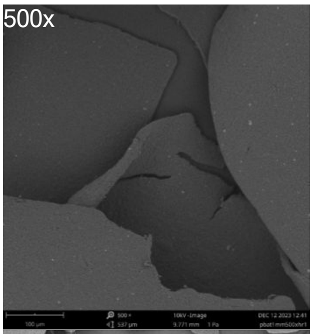	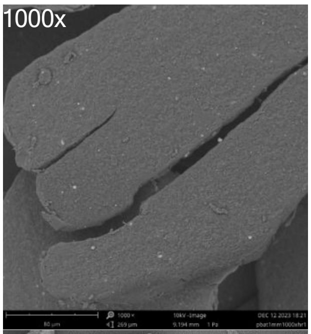
PBAT<br>75µm	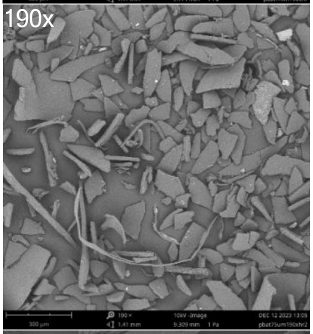	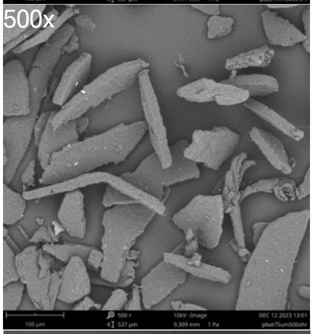	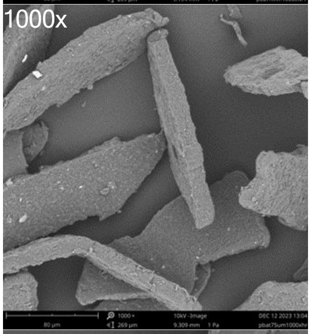
PE<br>1mm	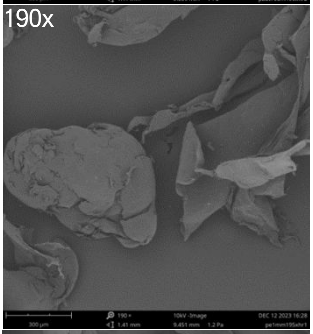	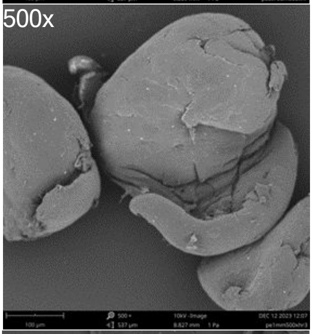	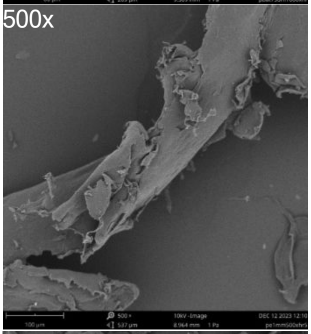
PE<br>75µm	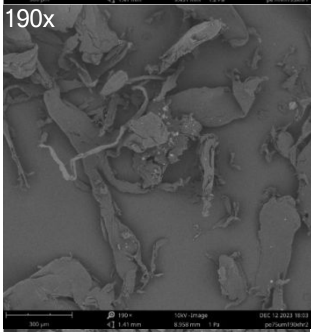	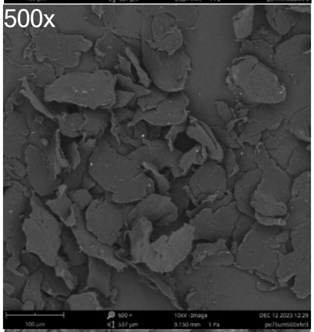	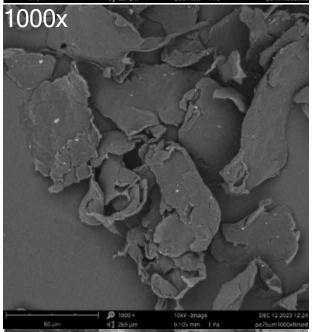
PLA<br>75µm	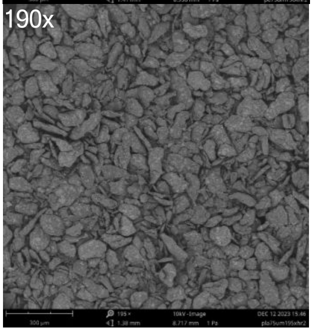	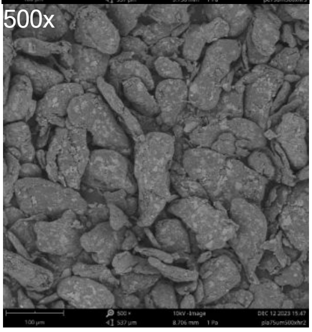	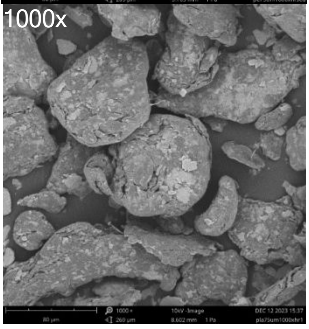
PS<br>75µm	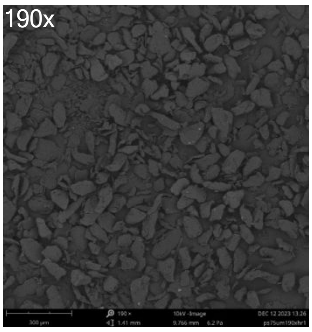	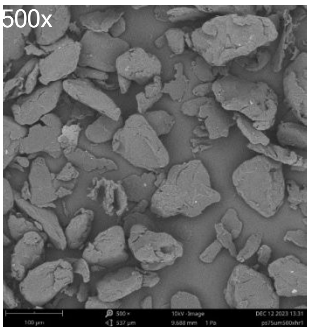	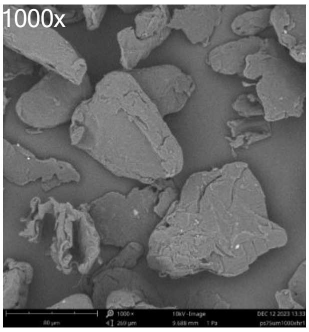

Table S3: Summary of the systematic exploration of the preprocessing steps and PCA scores plot. In the PCA scores plot the samples the convex hull includes samples of the same polymer: PBAT (black), PE (red), PLA (green), PS (blue).

Preproc. #	Baseline	Scattering	Smoothing	PCA (PC1 vs. PC2) scores plot
1	no	yes	no	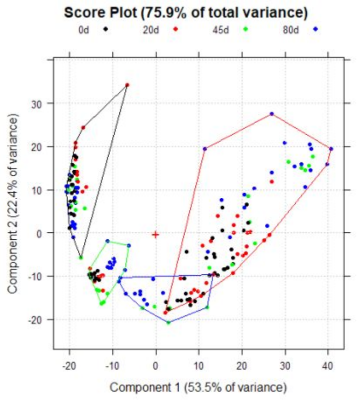
2	yes	no	no	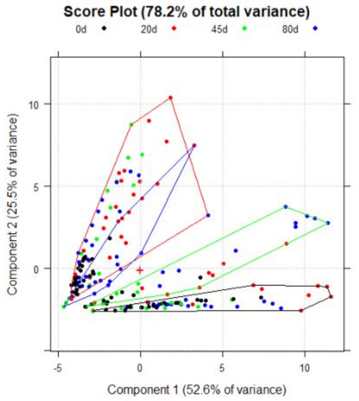
3	yes	yes	no	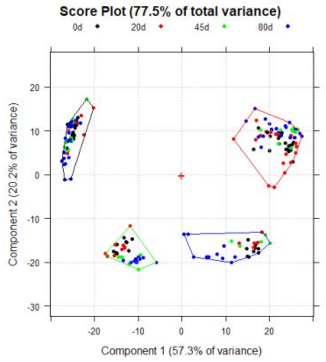
4	yes	yes	yes	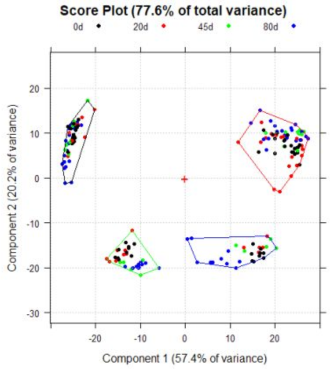

**5**	**no**	**yes**	**yes**	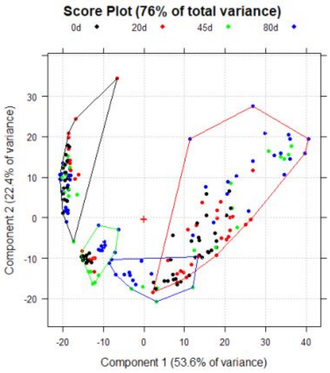
**6**	**yes**	**no**	**yes**	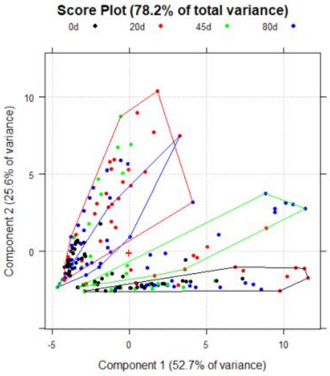

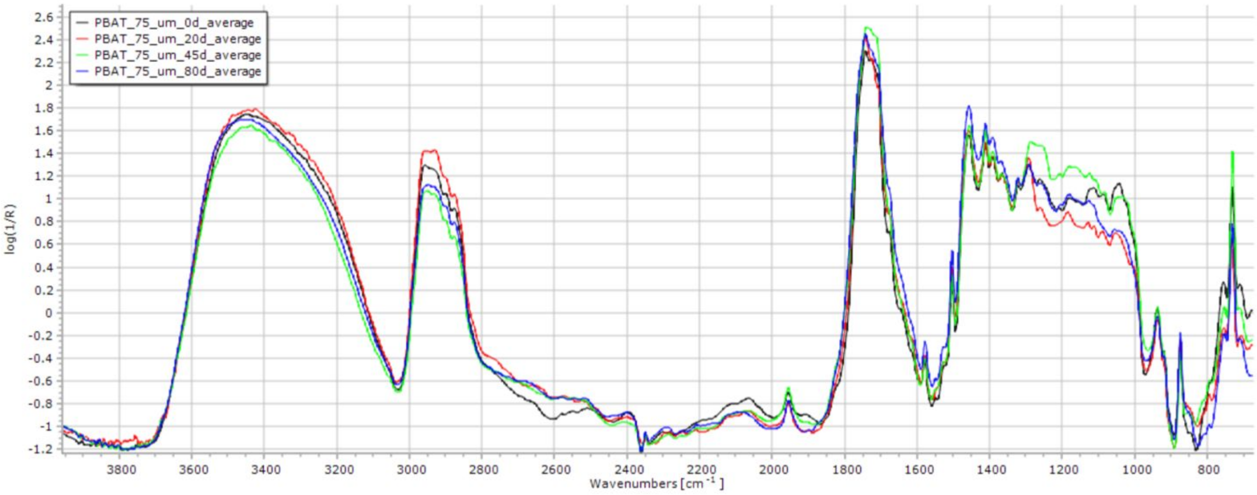

Figure S3: Averaged reflectance micro-IR spectra of PBAT particles at different aging times (black = 0 days, red = 20 days, green = 45 days, blue = 80 days).

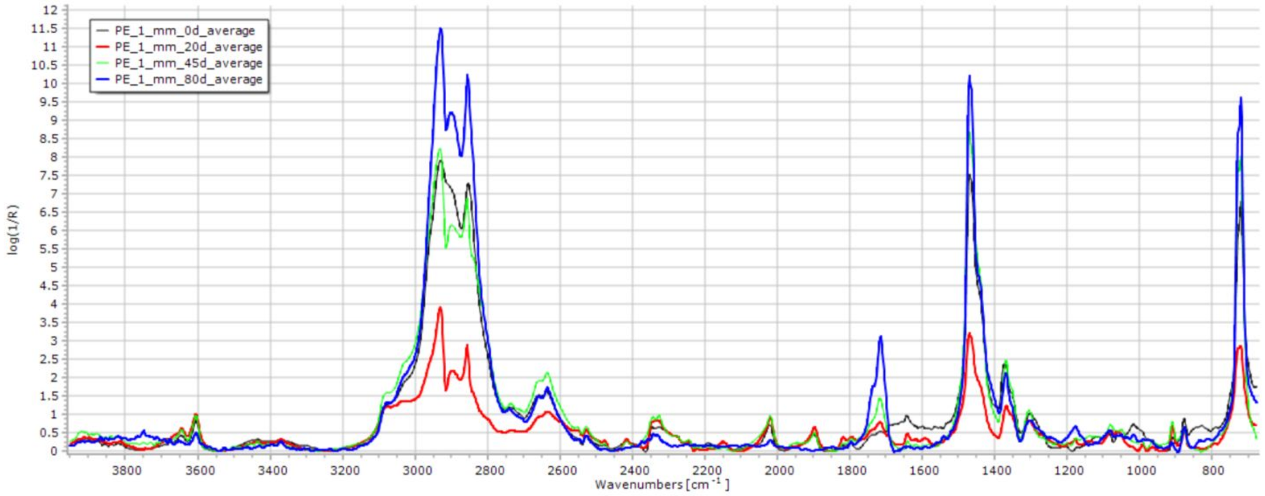

Figure S4: Averaged reflectance micro-IR spectra of PE particles sized <1mm, at different aging times (black = 0 days, red = 20 days, green = 45 days, blue = 80 days).

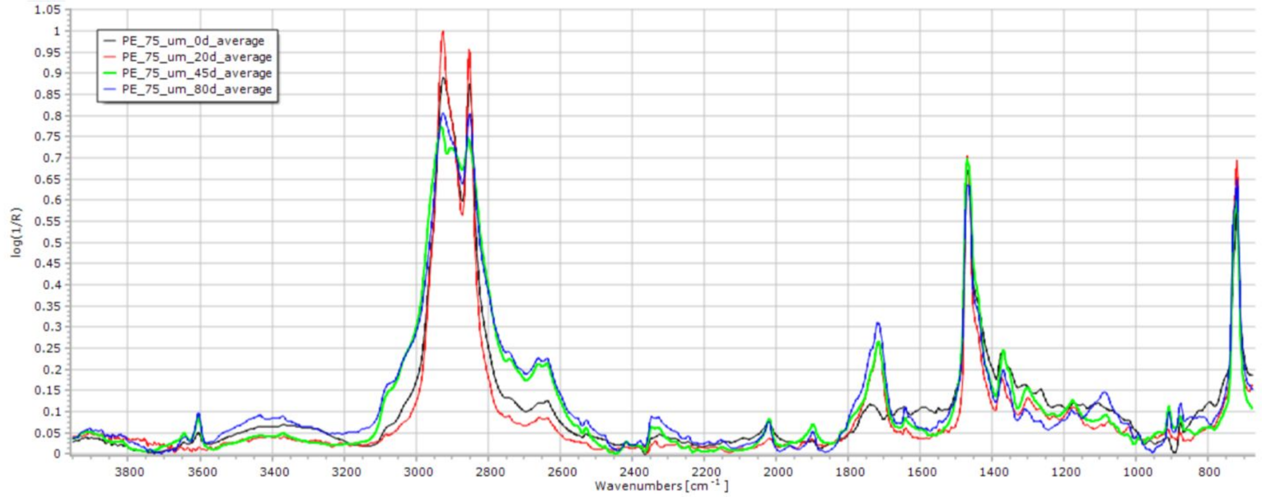

Figure S5: Averaged reflectance micro-IR spectra of PE particles sized <75um at different aging times (black = 0 days, red = 20 days, green = 45 days, blue = 80 days).

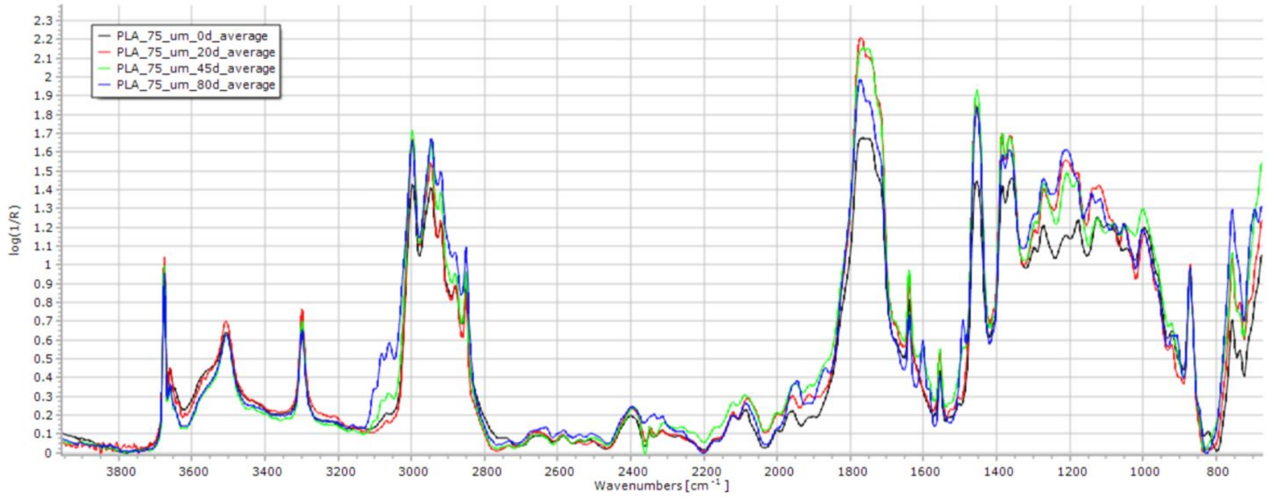

Figure S6: Averaged reflectance micro-IR spectra of PLA particles at different aging times (black = 0 days, red = 20 days, green = 45 days, blue = 80 days).

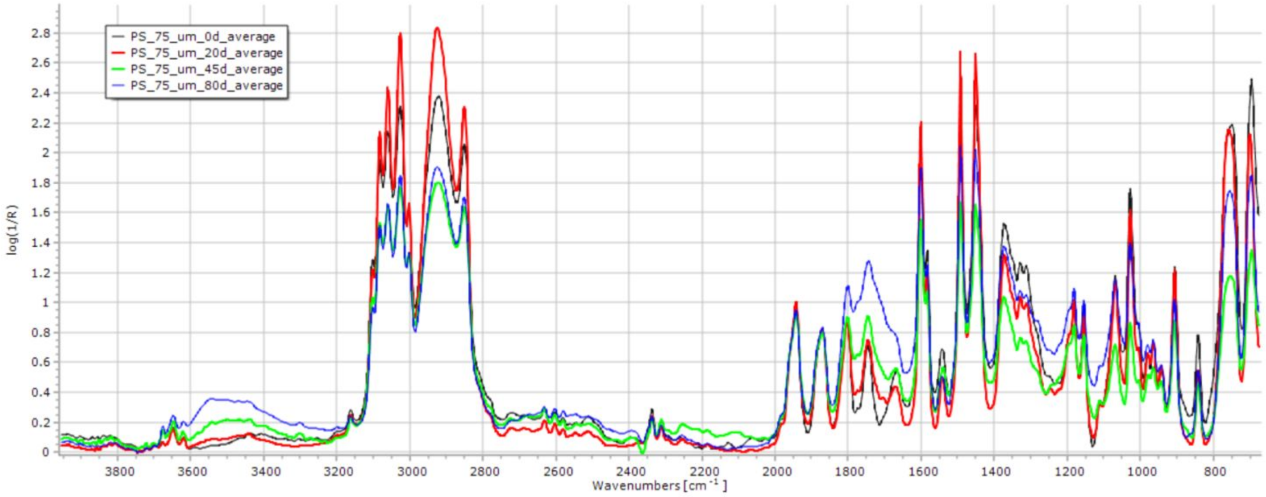

Figure S7: Averaged reflectance micro-IR spectra of PS particles at different aging times (black = 0 days, red = 20 days, green = 45 days, blue = 80 days).

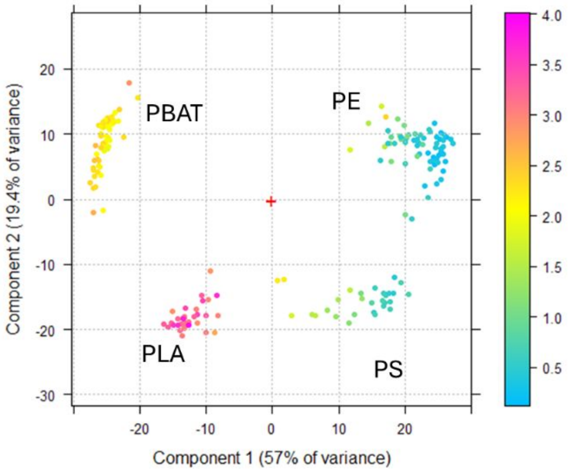

Figure S8: Scores plot of the PCA over the MPs dataset brushed with the CI values (scale on the right) calculated according to the SAUB method.

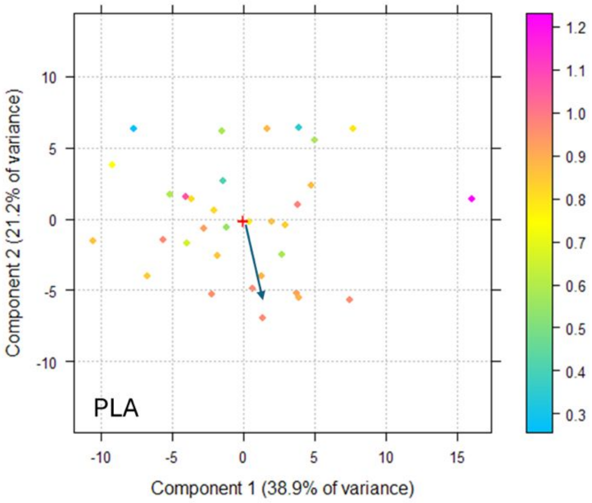

Figure S9: Scores plot of PC1 vs. PC2 from the PCA of PLA, brushed according to the ratio between the area of the band at 755 cm^{-1} versus the band at 860 cm^{-1}. The arrow points in the direction of the 755cm^{-1} loadings (cfr. Figure 4).
